# A multi-scale approach to study biochemical and biophysical aspects of resveratrol on diesel exhaust particle-human primary lung cell interaction

**DOI:** 10.1038/s41598-019-54552-w

**Published:** 2019-12-03

**Authors:** Wei Zhang, Qifei Li, Mingjie Tang, Han Zhang, Xiaoping Sun, Sige Zou, Judy L. Jensen, Theodore G. Liou, Anhong Zhou

**Affiliations:** 1 Department of Biological Engineering, Utah State University, 4105 Old Main Hill, Logan, UT 84322 USA; 2 Translational Gerontology Branch, National Institute on Aging, National Institutes of Health (NIH), Baltimore, MD 21224 USA; 3 Division of Respiratory, Critical Care and Occupational Pulmonary Medicine, Department of Internal Medicine, School of Medicine, University of Utah, Salt Lake City, UT 84132 USA

**Keywords:** Raman spectroscopy, Biomedical engineering

## Abstract

Diesel exhaust particles (DEPs) are major air pollutants that lead to numerous human disorders, especially pulmonary diseases, partly through the induction of oxidative stress. Resveratrol is a polyphenol that ameliorates the production of reactive oxygen species (ROS) and delays aging-related processes. Herein we studied the cytoprotective effect of resveratrol on DEP-exposed human lung cells in a factorial experimental design. This work investigates biophysical features including cellular compositions and biomechanical properties, which were measured at the single-cell level using confocal Raman microspectroscopy (RM) and atomic force microscopy (AFM), respectively. Principal component analysis (PCA), hierarchical cluster analysis (HCA) and partial least square regression (PLS) analysis were applied to analyze Raman spectra with and without resveratrol protection. The health status of individual cells could be effectively predicted using an index derived from characteristic Raman spectral peak (e.g., 1006 cm^−1^) based on PLS model. AFM measurements indicated that cellular adhesion force was greatly reduced, while Young’s modulus was highly elevated in resveratrol treated DEP-exposed cells. Anti-oxidant resveratrol reduced DEP-induced ROS production and suppressed releases of several cytokines and chemokines. These findings suggest resveratrol may enhance resistance of human lung cells (e.g., SAEC) to air pollutants (e.g. DEPs).

## Introduction

Poor air quality affects 92% of the world’s population. In some areas, people live with daily or even continuous exposure to air pollution levels well above WHO limits^1^. Diesel exhaust particles (DEPs), as one of common and major constituents of air pollution^2^, have a carbon core which absorbs mixtures of chemicals (polycyclic aromatic hydrocarbons, sulfate, nitrate, and heavy metals). DEPs are associated with numerous human diseases and disorders, such as cardiovascular disease^3^, lung cancer^4^, vascular dysfunction that leads to thromboembolic disease and hypertension^5^. In the lung, DEPs increase airway inflammation^6^, induce bronchial asthma, increase bacterial infection, heighten endotoxin-related alteration of cytokine profile, and alter gene regulation^7,8^. At the cellular level, 10 μg ml^−1^ DEPs can substantially increase the production of reactive oxygen species (ROS) and reduce mitochondrial activity^9^, which can induce oxidative damage to DNA, proteins and lipids and consequently lead to cell death via apoptosis^10^.

Respiration starts with air inhalation via the nose or mouth followed by mass transport through the trachea and bronchial passages to smaller and smaller airways ending in respiratory bronchioles. Gases diffuse from respiratory bronchioles to balloon-like air sacs named alveoli, which are the primary sites of gas exchange with blood. SAEC are isolated from the distal portion of the respiratory tract with most originating from alveoli. These cells are particularly suitable for investigating the toxicity of DEPs^11^, because they are derived from structures often involved early in many human diseases of high impact to populations, individuals or both.

Resveratrol (3,5,4′-trihydroxystilbene) (RES) is a naturally occurring phenolic compound found in red grapes, berries, knotweed, peanuts, and other plants^12^. RES exhibits anti-oxidant, anti-inflammatory, cardio-protective and anti-tumor activities, and may have effects against age-related diseases^13^, such as cancer, diabetes, and cardiovascular and neurological diseases^14^. RES interacts with a number of proteins and influences various signaling pathways involved in oxidative stress response and aging^12^. RES can activate the sirtuin family of proteins, most notably sirtuin 1 (SIRT1), partly via AMP-activated protein kinase (AMPK)^15^. The activation of SIRT1 can increase mitochondrial activity, improve mitochondrial aerobic capacity, and promote oxidative dephosphorylation^16^. On a larger scale, SIRT1 can function as a suppressor of PM-induced thrombosis^17^. AMPK is a serine/threonine protein kinase that senses cellular AMP/ATP levels and modulates metabolic homeostasis. AMPK is involved in numerous cellular processes, including glucose uptake, autophagy and anti-oxidant response^18^. RES exerts antioxidant and cardioprotective effects partly through the activation of the nuclear factor erythroid 2-related factor 2 (Nrf2)/ antioxidant response element (ARE) signaling pathway^19,20^. Nrf2 activity is associated with traffic-related pollution at the promoter transcription level^21^. RES directly increases cell proliferation at low concentration (5 μM), while inducing cell apoptosis at higher concentration (15 μM or more)^22^. Altogether, these effects suggest that RES may protect against the damaging effect of DEP-induced diseases. The damaging effect of DEPs on gene expression level and cytokine profile have been well studied^23,24^, but little attention has been paid to the study of protective effect of RES against DEPs^25^.

Stimulation by external toxic substances (like DEPs) may alter the function and structure of exposed cells. Simultaneous with DEP-cell interactions, chemicals adsorbed to DEPs can generate mechanical stress, resulting in changes in cell membrane integrity, deformation of cell structure, and changes in surrounding extracellular matrix^26^. Interaction with DEPs can alter the composition of cell surface molecules^27^, such as proteins, lipids, carbohydrates, nucleic acids, and others. Unfortunately, characteristic changes, including biophysical properties, cell biomechanics and cellular components, associated with DEP exposure and the effects of protective antioxidant activity require further investigation. Fluorescence staining techniques are commonly used in cytotoxicity studies^28^. But staining and fixation of cells before observation may alter or obscure intrinsic sample properties^29^. Atomic force microscopy (AFM) is based on the interaction between a sharp moving tip and the intact surface of a biological sample to visualize high-resolution topography on the nanometer scale. It can quantitatively measure adhesive force and Young’s modulus to potentially reveal otherwise unobservable links between biomechanics and human diseases^30^. AFM has been applied to quantitatively analyze drug-induced cytotoxicity on single tumor cells^31^. A complementary method, Raman microspectroscopy (RM), non-destructively measures vibration and rotational modes of macromolecules at the single-cell level using inelastic laser light scattering. RM identified the toxicity fingerprint of nanoparticle exposure on A549 cells in a time and dose dependent relation^32^. Both non-invasive, *in situ* techniques can measure cellular behaviors under near physiological conditions with high sensitivity, resolution and reliability making them suitable for studying healthy and pathological cells at the sub-cellular level^33,34^. Our lab previously applied AFM and RM together to study the cytotoxicity of DEPs on human normal and carcinoma cells^35,36^. These work demonstrated the feasibility of using these two label-free techniques as the novel tools to evaluate biomechanical and cellular properties of the cells exposed to toxic air pollutants.

In this study, we utilized AFM and RM to investigate *in vitro* cytoprotective effect of RES on human primary cells (SAEC) from interaction of DEPs at single cell level. We supplemented the effort with conventional methods including western blot and flow cytometry analysis to discover a wide range of cellular responses to DEPs exposure.

## Results

### RES attenuated cellular alterations of DEP-treated SAEC by RM

**Figure 1:**
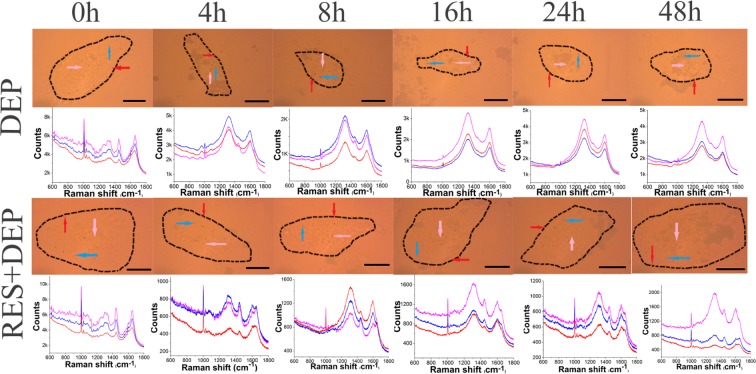
Light images and corresponding averaged Raman spectra of single SAEC treated with DEPs for different time periods in the absence or presence of RES. Confocal Raman spectra of SAEC taken at different cellular locations are denoted arrows of different colors: nucleus (pink), cytoplasm (blue) and cell membrane (red). Sixteen spectra (four points per location and four cells) were used to calculate the average spectrum for each location.

We characterized DEP-induced cellular component changes with RM by determining the specific intensity of spectral peaks over 48 h at the single cell level. Multiple chemometrics methods were also carried out to analyze the Raman data or establish predicting model. Light images and averaged Raman spectra of the cells treated with and without 10 μM RES are shown in Fig. 1. The Raman spectra at three locations per cell are plotted below an image showing corresponding locations in each cell identified by arrows: cell membrane (red), cytoplasm (blue), and nucleus (pink). Generally, more spectral peaks are observed at different time points in RES + DEP group, compared to DEP group, such as amide I (1660 cm^−1^), lipid (1451 cm^−1^), phenylalanine (1006 cm^−1^), DNA (786 cm^−1^) and tryptophan (1608 cm^−1^).

Principal component analysis (PCA) was applied to the original spectra to extract key information. In all following cases, the first two principal components (PCs) explained over 90% of the variance of the original data set. PCA plots of entire data set show two major spectra clusters (0 h versus other time points) regardless of RES pretreatment (Fig. S1). The results indicate damage effect of DEPs on SAEC that are different but not prevented with pretreatment of RES. After discarding outliers, score plots between DEP and RES + DEP group at different time points (Fig. S2) show tighter clustering of RES + DEP group principal component scores and more dispersed and displaced plots of DEP group. The two clusters are clearly separated at 0 h, but partially overlapped at other time points, due to highly scattered DEP plots. However, the hierarchical cluster analysis (HCA, in form of dendrogram) results in two main clusters, one refers to DEP group and the other corresponds to RES + DEP group. The clusters show a clear distinction between two groups except 0 h, indicating the similarity of original cell status before exposure to DEPs.

**Figure 2:**
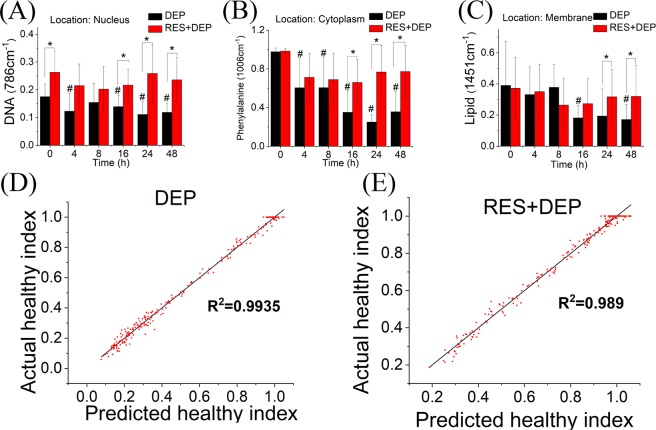
Barplot of characteristic peak at different cellular locations and PLS model using 1006 cm^−1^ as spectral predictor. (**A**) DNA at nucleus (786 cm^−1^), (**B**) phenylalanine at cytoplasm (1006 cm^−1^), (**C**) lipid at membrane (1451 cm^−1^). (**D**) PLS model calculated from Raman data (240 spectra) of DEP group, (**E**) PLS model calculated from Raman data (240 spectra) of RES + DEP groups. All peak intensities are averaged after baseline correction and normalization in absence (black bar) or presence (red bar) of 10 µM RES before exposed to 10 µg ml^−1^ DEP. Error bars were standard deviation. *Means significant difference between DEP and RES + DEP groups at each time point (P < 0.05). ^#^Means significant difference between DEP 0 h group to other DEP groups (4, 8, 16, 24, 48 h).

The alterations of characteristic peak intensity (after spectral data preprocessed by baseline correction and normalization) i.e. lipid (1451 cm^−1^), phenylalanine (1006 cm^−1^) and DNA (786 cm^−1^) at different cellular locations are plotted in Fig. 2A–C. The spectra at each cellular location was recorded after confocal laser illumination (arrows in Fig. 1). Peak intensity analysis first found that the damage effect varied with cell location. In the nucleus, the DNA peak ratio decreased by 22% from 0.18 at 0 hr to 0.14 at 16 hrs (Fig. 2A). In the cytoplasm, the phenylalanine peak decreased by 64% from 0.98 to 0.35 during first 16 h (Fig. 2B). At the cell membrane, the lipid peak decreased by 55% from 0.40 to 0.18 over the first 16 hours of exposure (Fig. 2C). Second, the peak intensity analysis found higher intensities for all molecules in the presence of RES regardless of the cellular locations, compared to DEP alone (Fig. 2). The peaks appear to recover after 8 hours of exposure for the RES + DEP conditions. Taking an example of 48 h exposure, the peak intensity in presence of RES were elevated significantly from 0.12 (DEP) to 0.24 (RES + DEP) at nucleus, 0.36 (DEP) to 0.77 (RES + DEP) at cytoplasm, and 0.17 (DEP) to 0.32 (RES + DEP) at membrane.

Partial least squares (PLS) models here presented (Fig. 2D,E) were developed by the combinations of spectra from different time points in two groups. Phenylalanine (1006 cm^−1^, Fig. 2D,E), lipid (1451 cm^−1^, Fig. S3) and Amide I (1660 cm^−1^, Fig. S4) peaks as well as peak ratio (1006 cm^−1^/1608 cm^−1^, Fig. S5) were employed as the marker peak for “healthy index”, which is the predictor in PLS model. ROS was also employed as the response in developing PLS model (Fig. S6). Both Fig. 2D,E show excellent fitting of linear regression plots (R^2^ = 0.9935 in DEP group and R^2^ = 0.989 in RES + DEP group) of the predicted healthy index (PHI) versus the actual healthy index (AHI). In the DEP group, the PLS model reduced the number of factors to six in total with minimum root mean PRESS (Prediction Residual Sum of Squares) of 0.22797. The cumulative variation explained by X and Y for the first factor was ~70% and ~99% respectively, which are similar to the reported value^37^. The PLS model of RES + DEP group showed 7 factors in total with minimum root mean PRESS of 0.33301. The cumulative variation explained by X and Y for the first factor was ~64% and ~99% respectively. The models were further verified with a group of testing spectra (e.g., eight spectra were used in Table 1), showing negligible differences between predicted and actual values in any particular time point. For instance, at 0 h, the PHI and AHI were 0.91 ± 0.10 and 0.90 ± 0.13 in DEP group, 0.79 ± 0.07 and 0.89 ± 0.07 in RES + DEP group, respectively. Overall, the AHI and PHI values in both DEP and RES + DEP groups show a similar decreasing trend from 0 hr to 48 hrs; however, the amplitudes of the decreases are different: AHI values in DEP group changed from 0.9 at 0 hr to 0.13 at 48 hr, a reduction of 0.77. RES pretreatment lead to a change from 0.89 to 0.54, or a reduction of only 0.35. It is worth noting that the healthy index in RES + DEP group present a “recovery” turnover point at 16 h (AHI 0.43, PHI 0.45), suggesting the protective effect of RES seen from PLS predicting model. Hence, our PLS model predicts the status of cells when exposed to DEPs simply by Raman spectra.

**Table 1:** Validation of PLS model (at 1006 cm^−1^) for DEP and RES + DEP groups using a group of testing Raman data (8 spectra were randomly selected from each time point).

Time (h)	DEP group		RES + DEP group	
	AHI	PHI	AHI	PHI
0	0.90 ± 0.13	0.91 ± 0.10	0.89 ± 0.07	0.79 ± 0.07
4	0.76 ± 0.21	0.77 ± 0.24	0.70 ± 0.32	0.75 ± 0.34
8	0.52 ± 0.24	0.50 ± 0.20	0.74 ± 0.33	0.72 ± 0.31
16	0.27 ± 0.15	0.32 ± 0.13	0.43 ± 0.28	0.45 ± 0.31
24	0.19 ± 0.16	0.20 ± 0.15	0.69 ± 0.31	0.70 ± 0.29
48	0.13 ± 0.03	0.15 ± 0.04	0.54 ± 0.24	0.65 ± 0.32

Data are shown as mean ± SD. Note: AHI means actual healthy index, PHI means predicted healthy index.

### RES attenuated biomechanical properties alterations of DEP-treated SAEC by AFM

**Figure 3:**
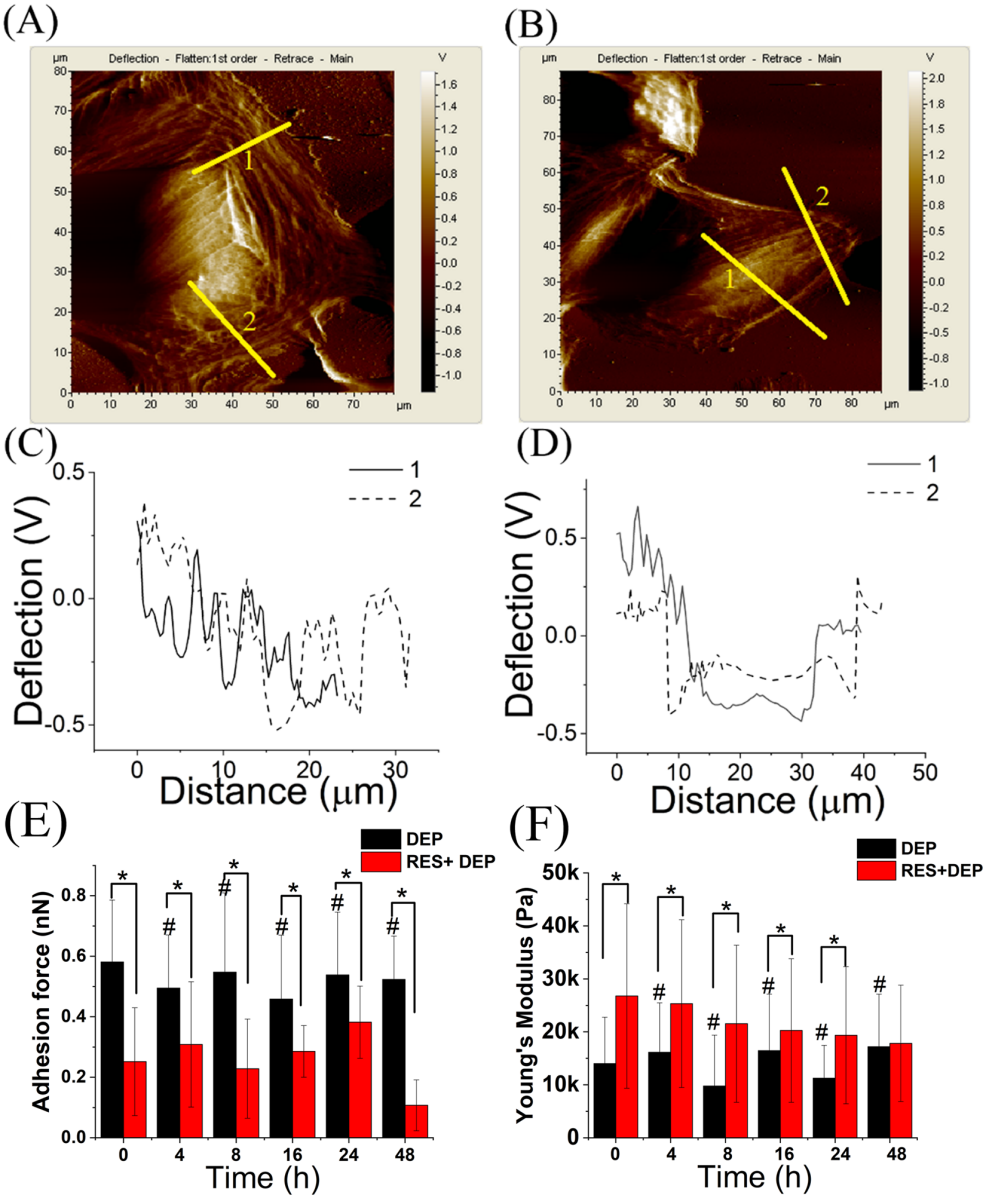
Representative AFM images and analysis of corresponding deflection profile and mechanical properties. (**A**) Deflection image of DEP group; (**B**) Deflection image of RES + DEP group; (**C**) deflection profile noted in (**A**); (**D**) deflection profile noted in (**B**); (**E**) barplots of adhesion force; (**F**) barplots of Young’s modulus. The data were obtained from multiple individual cells (N = 17 for each group, and 30 datum points on each cell). T-test was used to analyze the statistical difference between groups. *Means significant difference between DEP and RES + DEP groups at each time point (P < 0.05). ^#^Means significant difference between DEP 0 h group to other DEP groups (4, 8, 16, 24, 48 h).

AFM was used to quantify mechanical property changes (including membrane adhesive force and cell elasticity) of SAEC in culture medium. Representative AFM images of single cells with or without RES pretreatment are shown in Fig. 3A,B. Compared to the DEP group, more filamentous structures are visualized in the RES + DEP group (Fig. 3C,D). Furthermore, alterations of adhesive force and cell elasticity obtained from multiple cells under different exposure times are illustrated in Fig. 3E,F. The bar plots demonstrated that RES treatment generally reduced adhesive forces, reaching statistically significant reductions at 0, 8, 16 and 48 hour exposure time points, compared between DEP and RES + DEP groups. Reductions after 4 and 24 hour exposures to DEP were similar but did not reach statistical significance. Young’s modulus was increased in RES + DEP group compared to DEP group, but the difference became smaller with DEP exposure time and was no longer statistically significant after 8 hours of DEP exposure. The cells became stiffer (larger Young’s modulus) and more difficult to deform after RES pretreatment, suggesting an intact cytoskeletal structure, which is confirmed by fluorescence images (Fig. S7).

### RES attenuated ROS of DEP-treated SAEC

**Figure 4:**
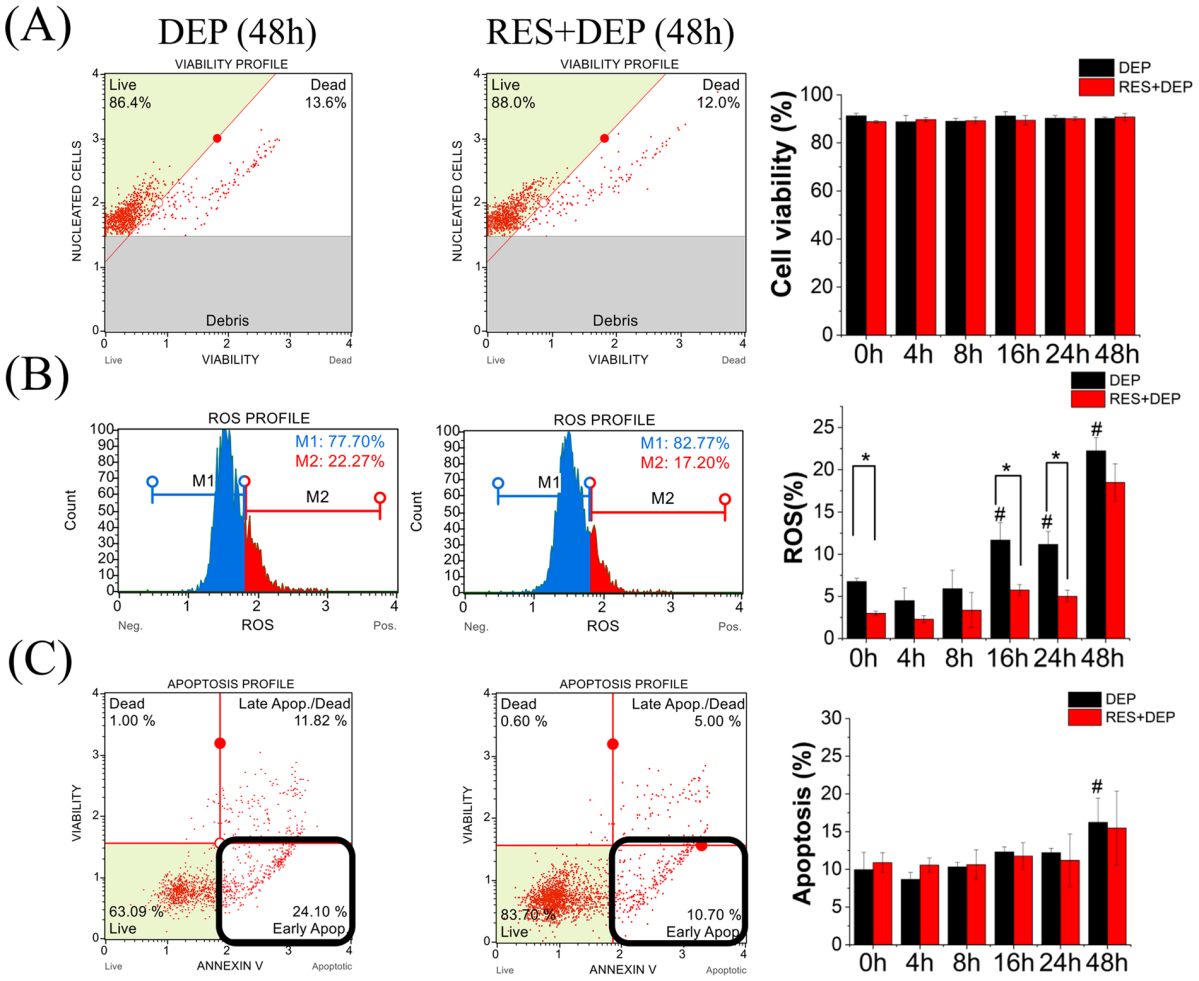
Cellular response to exposure to DEPs (10 µg ml^−1^) with or without pretreatment of RES (10 μM). Left two columns are representative cellular profile of each analysis (48 h as example). (**A**) Cell viability; (**B**) ROS, M1 (%) means cell percentage without ROS, M2 (%) means cell percentage with ROS; (**C**) Apoptosis, early apoptosis percentage (bottom right corner) was used. All data were collected with three repeats. Error bar is standard deviation (SD). *Means significant difference between DEP and RES + DEP groups at each time point (P < 0.05). ^#^Means significant difference between DEP 0 h group to other DEP groups (4, 8, 16, 24, 48 h).

Viability, ROS and apoptosis in different exposure groups are shown in Fig. 4 (representative cell responsive profiles at 48 hrs in left column). Cell viability was at least 90% in all treatment conditions, implying that DEPs at 10 µg ml^−1^ are not generally lethal within 48 h (Fig. 4A). However, Raman spectroscopy measurement shows its advantage over the cell viability analysis (conventional assay) to assess the DEP-induced cytotoxicity. The increase of ROS production began at 16 h and peaks at 48 h in DEP group. In terms of RES + DEP group, ROS increase did not occur until the first 24 h of exposure but appeared to rapidly elevate to nearly the similar level of ROS production with DEP group at 48 h (Fig. 4B). Apoptosis percentage of total cells gradually increased from 10% to 15% in a time dependent manner in both groups with no significant difference between DEP and RES + DEP groups at any time point (Fig. 4C). More importantly, compared to the DEP group, ROS productions were attenuated at each time point in the RES + DEP group.

### RES induces alteration of cytokine profile in DEP-treated SAEC by cytokine assay

**Figure 5:**
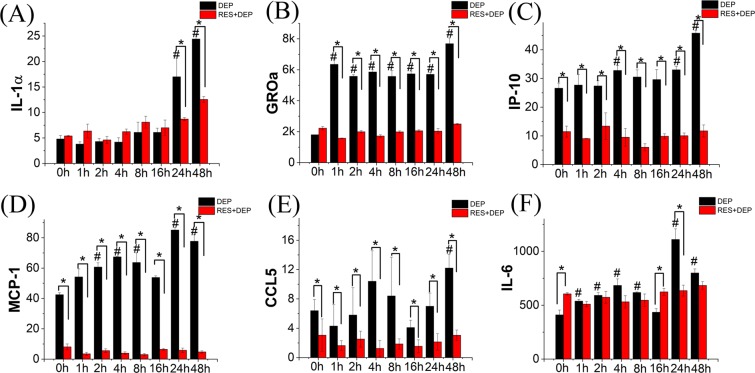
Barplots of cytokines and chemokines released from DEP induced SAEC with (red bar) or without (black bar) RES pretreatment. Unit of Y-axis: ng ml^−1^. Error bars are standard deviation of the mean. *Means significant difference between DEP and RES + DEP groups at each time point (P < 0.05). ^#^Means significant difference between DEP 0 h group to other DEP groups (4, 8, 16, 24, 48 h).

The secretion profiles of six cytokines and chemokines are shown in Fig. 5 (more are seen in Fig. S8). Briefly, both groups of cells had increases in IL-1α secretion as exposure times increased; however, RES prevented the near tripling of IL-1α secretion seen in cells treated only with DEPs at the 24 hours (Fig. 5A). Cells treated with RES in addition to DEP had about one-third the secretion of growth-regulated oncogene α (GROα), interferon gamma-induced protein 10 (IP-10), monocyte chemoattractant protein-1 (MCP-1) and chemokine ligand 5 (CCL5) compared to DEP alone at all time points (Fig. 5B–E). IL-6 expression levels fluctuated with a few time points showing significant difference between two groups over the 48 h exposure periods (Fig. 5F).

### Analysis of RES activated pathways on DEP-treated cell

**Figure 6:**
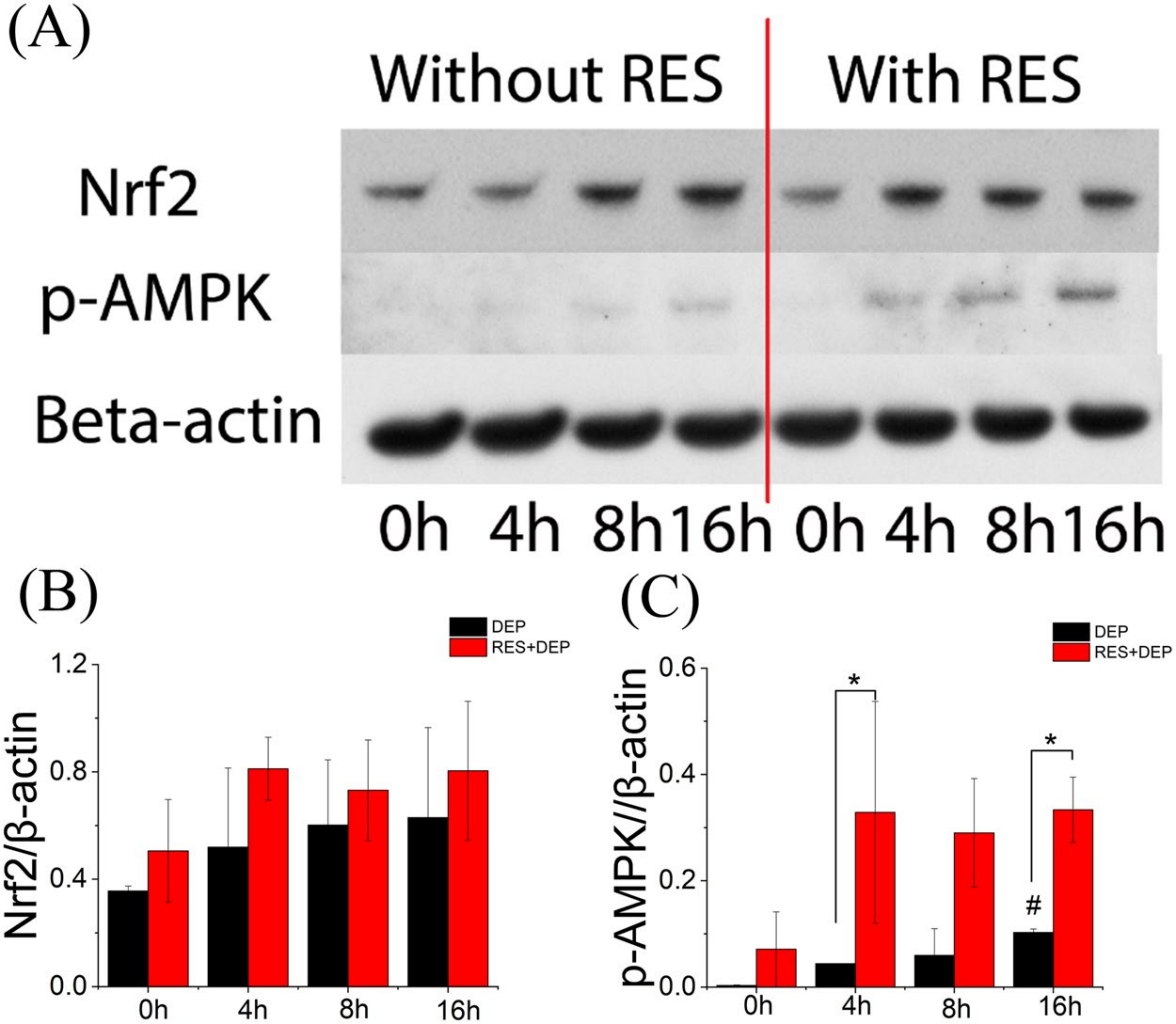
Representative western blot image and quantified barplots of Nrf2 and p-AMPK after exposure to DEPs alone or RES + DEPs. β-actin is used as internal control. Uncropped blots are presented in Supplementary Fig. S11. *Means significant difference between DEP and RES + DEP groups at each time point (P < 0.05). ^#^Means significant difference between DEP 0 h group to other DEP groups (4, 8, 16, 24, 48 h).

Western blotting of two proteins (Nrf2 and p-AMPK) shows a trend of gradually increasing of Nrf2 expression with DEP exposure in both groups (Fig. 6A,B). Pretreatment with RES for DEP-treated cells enhanced the expression levels of p-AMPK significantly after 4 and 16 h of exposure. (Fig. 6A,C).

## Discussion

**Figure 7:**
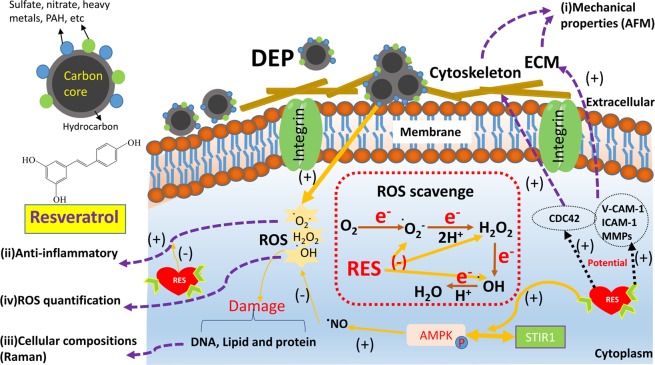
Schematic illustration of DEP-cell interaction mechanisms and RES protection on SAEC through intracellular oxidative stress signaling pathways.

Epidemiologic study shows DEPs are highly associated with cardiovascular disease and lung cancer^38^. Multiple cell lines including human lung cell lines BEAS-2B and A549 cells, human bronchial epithelial cells (HBEC3), human umbilical vein endothelial cells (HUVECs) have been studied to *in vitro* assess cytotoxicity of DEPs. The cytotoxic effects of DEPs in BEAS-2B and A549 cells were manifested as cell apoptosis, decreased protein concentrations, intracellular ROS production and increased expression of antioxidant genes^23^. The interaction of DEPs with HBEC3 led to changes in genes involved in metabolism of xenobiotics and lipids, as well as cytokine expression profile^24^. RES is a naturally occurring potent antioxidant that protects individual cells and tissues against environmentally-induced oxidative stress^12,39^ and may thus provide clinically relevant health benefits^14^. RES was found to increase nuclear respiratory factor-1 (NRF-1) transcription in an estrogen receptors (ER)-dependent manner in HUVECs, and ablated DEP inhibition of basal NRF-1 expression^25^. In a rat heart model, resveratrol exerted significant antioxidant and cardioprotective effects, possibly through the activation of the Nrf2/ARE signaling pathway^19^. Our current work substantially extends prior observations by examining effects on cell architecture on a micron scale to cellular composition on a molecular scale, to cell surface organization on a nanometer scale. A schematic summarizes the cell-DEP interaction mechanisms including DEP inducement, ROS generation, and activation of certain signaling factors in Fig. 7.

A number of different techniques were applied here to examine cell responses to external exposures (DEP or RES + DEP) over a time course of 0 to 48 hrs. Immediately after DEP addition (Fig. 7(i)), cell membrane acts as the first barrier against DEP, which quickly affects the adhesion force and Young’s modulus at 0 h (Fig. 3) and by changing the cytoskeleton and extracellular matrix (ECM) proteins. RES can induce cellular phenotype changes depending on intracellular calcium and tyrosine kinase activities, and assembly of actin microfilaments and microtubules^40^, possibly regulating on Cdc42 gene^41^. As a consequence, Young’s modulus in RES + DEP group was significantly elevated in first few hours of exposure (Fig. 3F). RES also downregulates intercellular adhesion molecule-1 (ICAM-1), vascular cell adhesion molecule-1 (VCAM-1)^42^, and inhibiting matrix metalloproteases (MMPs) secretion^43,44^, leading to the lower adhesion force in RES + DEP group (Fig. 3E). Second (Fig. 7(ii)), cells start to activate anti-oxidant signal pathway including Nrf2 and AMPK around 4 h exposure (Fig. 6). Activation of Nrf2 antioxidant signaling can attenuate NFκB mediated cytokine profile alteration^45^. The pretreatment of RES inhibits the activity of NFκB^46^, resulting in remarkable changes of cytokine profile in RES + DEP group (Fig. 5). The Nrf2 pathway was not effectively activated by DEP exposure (Fig. 6B), leading to substantial changes in cytokine markers including MCP-1, IP-10, GROα and CCL5 in DEP group throughout the entire time of period (Fig. 5). Third (Fig. 7(iii)), ROS generation began to significantly increase at 16 h without neutralization of RES. Meanwhile, Raman peak analysis (Fig. 2) shows a recovery trend with RES at 16 h exposure, reflecting the protective effect of RES at a molecular level. Fourth (Fig. 7(iv)), cell apoptosis begins at 48 h exposure (Fig. 4C). Within the initial 48 h period, cell death is not observed (Fig. 4A), probably due to the low concentration of DEPs. Therefore, the effect of RES on cell protection was sequentially observed by AFM (membrane mechanics), cytokine/chemokine assay (cytokine responses), Raman analysis (cellular composition) and other oxidative stress induced cellular responses (ROS and apoptosis). Within these quantitative measurements, our data on cell mechanics and components successfully demonstrated that AFM and Raman spectroscopy can effectively monitor the pathological states of the cells exposed to toxic DEP and reveal the extent of protection due to antioxidant pre-treatment, providing new insights on the biophysical responses on DEP-primary cell interaction activities.

PLS is a multivariate technique based on extracting latent variables, which maximizes covariance between the spectral variables and the variable to be predicted^37^. Different variables (spectral peaks, peak intensity ratio, and ROS) were tested as the predictor or response in our PLS model. Favorable AHI/PHI values were obtained when using the phenylalanine peak (1006 cm^−1^, Fig. 2/Table 1), or the lipid peak (1451 cm^−1^, Fig. S3/Table S1), or amide I peak (1660 cm^−1^, Fig. S4/Table S2) as the predictors. It is noted that the phenylalanine peak at 1006 cm^−1^ gave rise to the best PLS performance as their AHI and calculated /PHI values are monotonically decreasing with the increase in DEP exposure time (0 to 48 h), which makes biological sense because the cells are suffering oxidative damage in such exposure period (Table 1, Tables S1, S2). In terms of R^2^, AHI and calculated PHI values, none of the PLS models work well using the peak intensity ratio (1006 cm^−1^/1608 cm^−1^, Fig. S5/Table S3), as well as ROS (Fig. S6/Table S4) as the predictor. We also used a cell-based randomly selection method to separate training data and testing data (Fig. S9/Table S5), using the spectra collected from three of the four cell samples in each time point as training data (36 spectra) and one cell as testing data (12 spectra). The results show excellent performance of the prediction model but suggest that more cell samples are desirable for PLS analysis. Hence, RM combined with PLS analysis can be used as a powerful tool to non-invasively evaluate the healthy status of biological samples, especially in cytotoxicity.

We studied SAEC to ensure that observed *in vitro* responses to air pollution are closer to normal physiology than seen with the use of lung cancer cells (A549) as reported in our previous work^35^. Raman spectral peaks derived from human lung cells have been reported previously^47,48^ that allow monitoring of DNA, proteins, and lipids changes over time. For example, most cells present a characteristic Raman peak at 1006 cm^−1^ assigning the presence of phenylalanine^49,50^, which was selected as the spectral indicator to monitor cell behavior^51^. In the DEP group, phenylalanine peak intensity decreased significantly after 4 h DEP exposure, and dropped from 0.97 at 0 h to 0.35 at 16 h (Fig. 2B), due to oxidative damage. Meanwhile, ROS production remained consistent during the first 8 h and increased remarkably at 16 h in DEP group (Fig. 4B), which shows a high correlation between Raman analysis and ROS assay results, and underscores the sensitivity of Raman measurement for monitoring cell behaviors and cell processes. DEP exposed A549 cells had only a slight decrease of peak intensity at 786 and 1660 cm^−1^ during 0 h to 16 h, but SAEC peak intensity decreased by at least 22% and up to 64% for all molecules during the same exposure period, indicating both the sensitivity of SAEC to DEP damage and the marked difference between SAEC and a cancer cell line. These findings verify the protection effect of RES on different cellular locations at the compositional level by monitoring the changes of characteristic spectral peak associated with these cellular components.

High resolution AFM imaging of a single cell directly measures nanomechanical properties. The elastic properties of living cells are described by Young’s modulus defined as a measure of cellular deformability. Cancer cells generally have a lower Young’s modulus than normal cells^36^, which can be used as a biophysical marker to improve the diagnosis of cancer^52^. We evaluated the Young’s modulus in DEP exposed primary cells with or without RES protection. Figure 3(F) shows that the cells exposed to DEP have the fluctuating changes of Young’s modulus over 48 h, compared to that at 0 h. On the other hand, Young’s moduli measured in protected (RES + DEP) groups are statistically higher (P < 0.05) than those in exposed (DEP) group at each time point except 48 hr. Whereas the difference between protected and exposed groups at each time point is decreased from 12 kPa (0 h) to 0.6 kPa (48 h), which was closely related to the increased ROS levels in RES + DEP group at 48 hr (Fig. 4B). These findings suggest that Young’s modulus may serve as a biophysical marker sensing the beneficial effect of RES against DEP damage in early stage, but seems not sensitive enough in long term DEP/RES treatment. Furthermore, our study shows decreased adhesive force in primary cell (SAEC) with RES pretreatment, the opposite response compared to A549 cells^35^. As an example, after 48 h DEPs exposure, the addition of RES increases adhesive force from 0.6 to 0.7 nN in A549 cell, while this value was reduced from 0.52 to 0.10 nN in SAEC. These findings reinforce the implication that primary cells (SAEC) are more sensitive to DEPs than cancer cells and that use of cancer cells such as A549 may underestimate the damage caused by DEPs exposure.

Although the molecular mechanisms underlying such contrasting responses remain incompletely understood, our results are consistent with numerous prior findings. For example, RES induces apoptosis of cancerous cardiac HL1-NB cell but not normal cardiomyocytes^53^. Contrasting RES effects on gene expression of nicotinamide phosphoribosyltransferase, SIRT1 and plasminogen activator inhibitor 1 were similarly reported for hepatocarcinoma cells and primary human hepatocytes^54,55^. Numerous studies^56–58^ have demonstrated that RES can activate AMPK signaling pathway at least by increasing AMPK phosphorylation, which is consistent with our finding on p-AMKP level (Fig. 6). AMPK signaling has been implicated in various cellular stress responses. For example, AMPK activation can suppress mammalian target of rapamycin complex 1 (mTORC1), which results in increased autophagy, protein homeostasis and mitochondrial homeostasis^56^. The combinatory effects of these AMPK-mediated functions may underlie the longer survival of cells pre-treated with RES under DEP assault. The contrasting responses to RES on biomolecular levels, cellular mechanical properties and cytokine responses between SAEC and A549 may be related to differential regulation at the gene level.

In conclusion, a number of techniques were applied on multiple scales to explore potential mechanisms of the protective effect of RES against DEP-induced oxidative stress on human primary lung cells. Non-invasive AFM and RM achieve *in situ* measurements of membrane structures, cytoskeletal organization and cellular integrity, which are all closely correlated with the alterations at molecular or protein levels. The analysis of RM characteristic peak highlights the sensitivity of proteins, lipids and DNA in different cell locations to DEP induced damage and the overall protective effects on cells by RES. The development of a PLS model using a specific assigned Raman peak as predictor allows us to effectively predict the cell status when exposed to pollutants. Overall, our findings demonstrate systematic evidence of DEP-human primary cell interaction protected by RES at sub-cellular level. In future work, it would be particularly interesting to identify the cellular biomarkers for these multi-dimensional measurements: biophysical, biochemical, and gene expression, for the same exposed and protected cells; and examine whether responses would be dependent upon the DEP concentration (or chemical compositions) and exposure time, as well as antioxidant treatment conditions. Subsequently, an appropriate multi-scale computation model considering these parameters could be developed to describe cell activities reflecting cytotoxicity from DEP and cytoprotective effects of antioxidant treatment, which may facilitate the discovery of new pharmaceutical products protective against the air pollution.

## Methods and Materials

### Cell culture and treatment procedure

SAEC isolated from the distal portion of normal human lung tissue were purchased from Lonza (Anaheim, CA, USA), and cultured in small airway epithelial basal medium (SABM) supplemented with growth factors supplied in the SAGM SingleQuot® kit (Lonza) at 37 °C with 5% CO_2_ in a humidified atmosphere. EDX spectrum shows that DEPs consist of non-metal elements, metal and metal oxide compounds (major elements include Carbon, Chromium and Iron). DEPs (10 µg ml^−1^) were mixed with 2 mL culture medium and vortexed for 10 s, and subsequently sonicated for 20 min at room temperature. SAEC underwent pretreatment with plain medium or medium containing RES at 10 µM for 24 hours. SAEC were then treated with DEP solution at 10 µg ml^−1^ for 0 (control), 4, 8, 16, 24 or 48 hours. To minimize background noise for RM, cells at a density of 1 × 10^5^ per 2 mL of media were plated on cleaned magnesium fluoride (MgF_2_) optical windows (United Crystals Co., Port Washington, NY, USA). For AFM and confocal microscopy experiments, cells were seeded on poly-L-lysine coated glass bottom Petri dishes (MatTek Corp., Ashland, MA, USA). The morphology (SEM images) of cells with or without DEPs treatment are illustrated in Fig. S10. All methods were carried out in accordance with relevant guidelines and regulations.

### Raman microspectroscopy

The Raman spectra were measured by a Renishaw inVia Raman spectrometer (controlled by WiRE 3.3 software, Renishaw, UK) connected to a Leica microscope (Leica DMLM, Leica Microsystems, Buffalo Grove, IL, USA), equipped with a 785 nm near-infrared (IR) laser that was focused through a 63 × NA = 0.90 water immersion objective (Leica Microsystems, USA). After cells were pretreated with plain medium or medium containing RES (10 µM) for 24 h, DEPs were first introduced into the 48 hr exposure group (48 h prior to measurement), followed by the 24, 16, 8 and 4 hour exposure groups (hours mean the time prior to measurement, respectively) with the control (0 hour) group last. DEPs are gently washed away from the cells before Raman measurements. All spectra were collected at the same time (within 30 min). Three different locations (nucleus, cytoplasm and membrane) on each cell were measured. Sixteen spectra (four points per location and four cells) were baseline corrected using asymmetric least squares smoothing method (asymmetric factor 0.001, threshold 0.05, smoothing factor 5, number of iterations10), and normalized before using chemometrics analysis including PCA, HCA and PLS in Origin 2018. In PLS model development, a few representative peaks (1006 cm^−1^, 1451 cm^−1^, 1660 cm^−1^) and peak ratio (1006 cm^−1^ to 1608 cm^−1^) were defined as the AHI. The remaining wavenumbers excluding selected peaks were served as independent variables. 40 spectra (48 spectra in total) in each time point were randomly selected as the training data. The remaining 8 spectra were used to test the PLS model.

### Atomic force microscopy

AFM measurements are referred to our previous paper^35,36^. Briefly, topography and deflection images are obtained in fixed cells (4% paraformaldehyde for 10 min). Cell elasticity and adhesive force were measured *in situ* without pretreatment. All measurements were accomplished within one hour in order to observe cells in conditions as close to normal physiology as possible. At least 20 cells for each condition and 15 force curves for each cell were collected to reduce the likelihood of spurious results.

### Muse cell analyzer

Cell viability, apoptosis and ROS were measured by Muse cell analyzer (EMD Millipore Corporation, US). SAEC were cultured in 12-well plates (Celltreat, Shirley, MA, US) and treated with DEPs using staggered starting times as mentioned above. Samples were centrifuged at 1000 × g for 5 minutes (Thermo Scientific CL2 centrifuge) and mixed with Muse agents (life science of Merck KGaA, Darmstadt, Germany) before testing by the analyzer (protocol provided by EMD Millipore Corporation). Each sample had three replicates. One-way ANOVA was performed via Origin 2018 to examine results from the study groups with P < 0.05 set as indicating a statistically significant difference. A series of t-tests (Origin 2018) were conducted to statistically analyze the difference between control (0 h DEP) and other time points in DEP group.

### Cytokine and chemokines release

To analyze the secretion of cytokines and chemokines by SAEC, cells were pretreated for 24 hours with RES and exposed to DEPs for 4, 8, 16, 24 and 48 hours. Cell supernatants were first centrifuged at 250 × g for 5 minutes to remove cell debris. Subsequently, supernatants were centrifuged at 2500 × g for 5 minutes to remove DEPs then frozen for storage at −80 °C. The samples were tested as a single batch on Quansys Biosciences’ (Logan, UT) Q-Plex^TM^ Array kits for human cytokines and chemokines. The data were reported as mean ± SD.

### Protein isolation and Western blot analysis

After DEP treatment as described above, protein was extracted from collected SAEC using RIPA buffer (Sigma-Aldrich, Cat. No. R0278) containing protease inhibitor cocktail set III (Calbiochem, Cat. No. 539134).

Protein was separated by electrophoresis with a Nupage gel and transferred to a PVDF membrane with the iBot Dry blotting system (Invitrogen). For Western blot analysis, proteins of interest were probed with specific primary antibodies against Nrf2 (Santa Cruz, Cat. No. sc-722), pAMPK (Cell Signaling, Cat. No. 2532 s), and β actin (Abcam, Cat. No. 8224) as an internal control, at appropriate dilution from 1000–5000. Secondary antibodies were horseradish peroxidase conjugated (HRP) goat anti-rabbit IgG (Abcam, Cat. No. ab6721) and HRP goat anti-mouse IgG (Abcam, Cat. No. ab6789), at a 5000–20,000 of dilution. The signals were detected with Amersham ECL Plus (GE Healthcare, Cat. No. RPN2132). Each treatment sample was repeated with at least three biological replicates. ImageJ (1.47 V) was used to quantify the band intensities by subtracting background signal of each band (underneath area). The real signal values of Nrf2 and p-AMPK in all groups were normalized by the intensity of β actin bands. The comparisons among each group were performed by one-way ANOVA using Origin 2018.

## Supplementary information

supplementary materials

## Data Availability

The datasets generated and/or evaluated during the current study are available from the corresponding author on request.
