# Supplementary material for: A multi-scale approach to study biochemical and biophysical aspects of resveratrol on diesel exhaust particle-human primary lung cell interaction
Source: Sci Rep. 2019 Dec 3;9:18178. doi: 10.1038/s41598-019-54552-w (PMC6890693; doi:10.1038/s41598-019-54552-w)
Supplement: Supplementary file 1 — supplementary materials [file 41598_2019_54552_MOESM1_ESM.pdf]

Supplementary information

A multi-scale approach to study biochemical and biophysical aspects of resveratrol on diesel exhaust particle-human primary lung cell interaction

Wei Zhang[1], Qifei Li[1], Mingjie Tang[1], Han Zhang[1], Xiaoping Sun[2], Sige Zou[2], Judy L. Jensen[3], Theodore G. Liou[3], Anhong Zhou[1,*]

[1]Department of Biological Engineering, Utah State University, 4105 Old Main Hill, Logan, UT 84322, U.S.A.

[2]Translational Gerontology Branch, National Institute on Aging, National Institutes of Health (NIH), Baltimore, MD 21224, U.S.A

[3]Division of Respiratory, Critical Care and Occupational Pulmonary Medicine, Department of Internal Medicine, School of Medicine, University of Utah, Salt Lake City, UT 84132, U.S.A.

***Corresponding to:**

Anhong Zhou, Ph.D. Professor

Department of Biological Engineering,

Utah State University, 4105 Old Main Hill, Logan, Utah 84322-4105, U.S.A.

Tel: 1-435-797-2863 (office); Fax: 1-435-797-1248

Email: Anhong.Zhou@usu.edu

Principal component analysis (PCA)

Original Raman data set (288 spectra in total for each group) excluding background spectra were classified into two main clusters by PCA in both DEP and RES+DEP group, one without DEP exposure and another one with different exposure time. The PC1 loading plot (Figure S1 C-D) show the peak positions at wavenumber of 1006, 1321, 1451, 1608, 1660 cm^{-1} have the highest weights for the PCA discrimination of different groups.

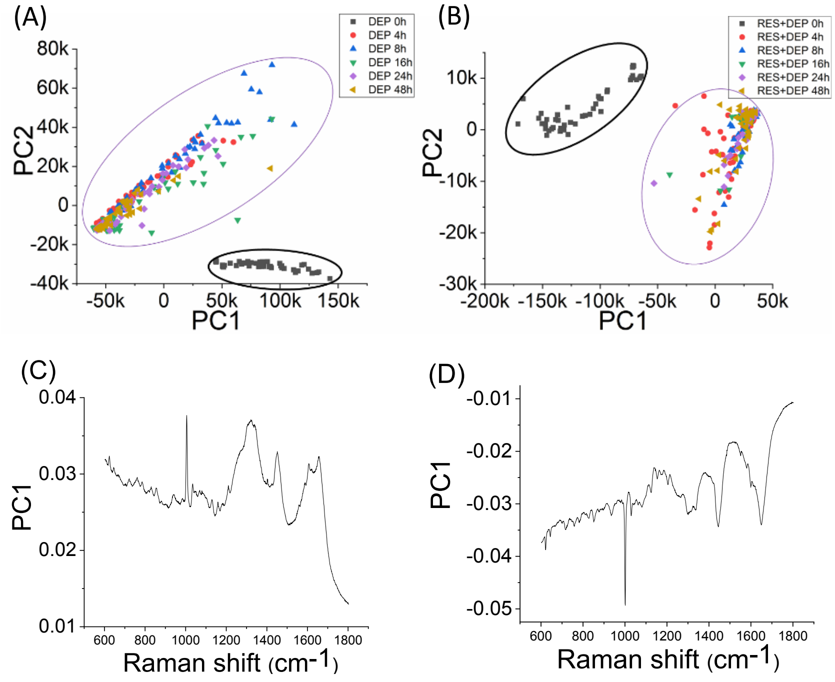

Figure S1: Principal component plots and loadings of whole Raman data despite of location. (A) DEP group; (B) RES+DEP group; (C) PC1 loading plot of DEP group; (D) PC1 loading plot of RES+DEP group

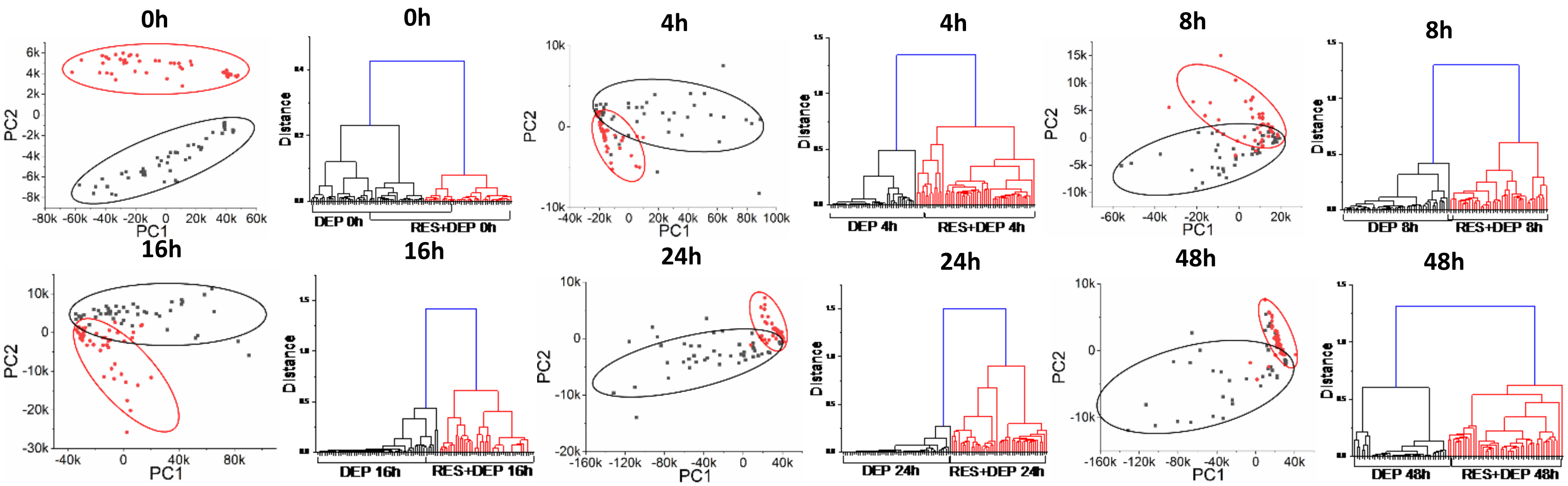

Figure S2: PCA score plots and dendrograms obtained by HCA between DEP (black) and RES+DEP (red) group in different exposure time. Black trees indicate DEP group, red trees indicate RES+DEP group.

Partial least square regression

Lipid (1451 cm^{-1}) peak were employed as the healthy index, which is the predictor in PLS model.

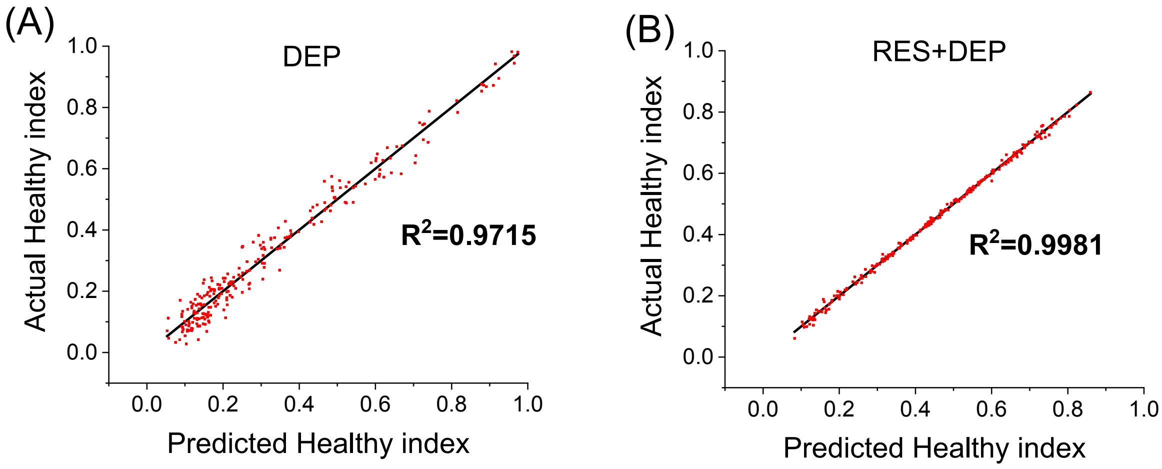

Figure S3: Partial Least square regression model of DEP (A) and RES+DEP (B) group, using lipid (1451cm^{-1}) intensity as healthy index (predictor) based on majority Raman data (240 spectra).

Table S1: Validation of PLS model using a small portion of Raman data (8 spectra of each time point)

	DEP group		RES+DEP group	
Time (h)	AHI	PHI	AHI	PHI
0	0.16±0.06	0.16±0.06	0.22±0.09	0.22±0.07
4	0.35±0.17	0.37±0.10	0.34±0.18	0.38±0.18
8	0.35±0.16	0.31±0.14	0.31±0.14	0.33±0.16
16	0.16±0.08	0.18±0.07	0.23±0.15	0.22±0.15
24	0.11±0.06	0.11±0.06	0.29±0.13	0.29±0.14
48	0.13±0.05	0.15±0.02	0.19±0.16	0.25±0.14

Note: AHI means actual healthy index, PHI means predicted healthy index

Amide I (1660 cm^{-1}) peak were employed as the healthy index, which is the predictor in PLS model.

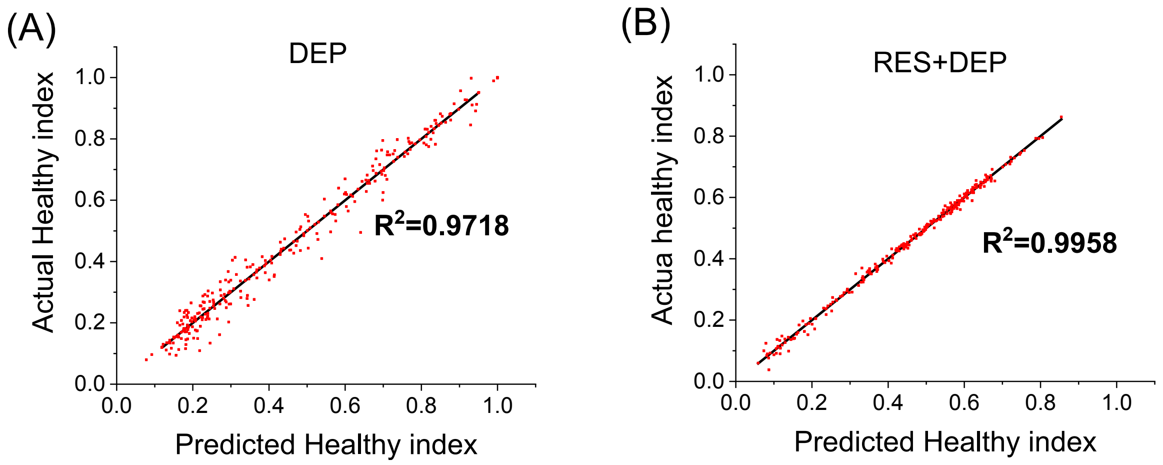

Figure S4: Partial Least square regression model of DEP (A) and RES+DEP (B) group, using Amide I (1660cm^{-1}) intensity as healthy index (predictor) based on majority Raman data (240 spectra).

Table S2: Validation of PLS model using a small portion of Raman data (8 spectra of each time point)

	DEP group		RES+DEP group	
Time (h)	AHI	PHI	AHI	PHI
0	0.47±0.16	0.49±0.14	0.22±0.09	0.23±0.08
4	0.59±0.10	0.59±0.14	0.41±0.26	0.42±0.25
8	0.49±0.21	0.48±0.19	0.38±0.27	0.41±0.23
16	0.25±0.11	0.29±0.11	0.25±0.19	0.25±0.20
24	0.19±0.07	0.21±0.07	0.33±0.20	0.30±0.17
48	0.15±0.05	0.18±0.03	0.27±0.15	0.28±0.20

Note: AHI means actual healthy index, PHI means predicted healthy index

Peak ratio of Phenylalanine (1006 cm^{-1}) versus tryptophan (1608 cm^{-1}) were employed as the healthy index, which is the predictor in PLS model.

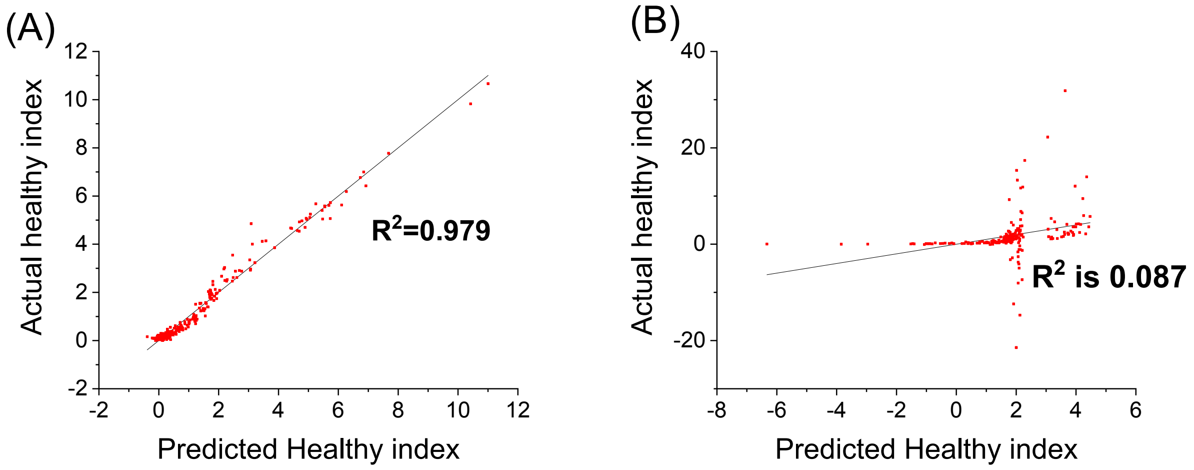

Figure S5: Partial Least square regression model of DEP (A) and RES+DEP (B) group, using Peak ratio of Phenylalanine (1006 cm^{-1}) versus tryptophan (1608 cm^{-1}) as healthy index (predictor) based on majority Raman data (240 spectra).

Table S3: Validation of PLS model using a small portion of Raman data (8 spectra of each time point)

	DEP group		RES+DEP group	
Time (h)	AHI	PHI	AHI	PHI
0	4.44±1.33	4.34±0.95	3.33±2.1	3.37±0.28
4	0.21±0.01	0.45±0.42	0.69±0.45	1.46±0.59
8	0.34±0.19	0.44±0.52	0.39±0.19	0.97±0.45
16	0.14±0.07	0.0027±0.50	0.62±0.68	0.69±0.84
24	0.14±0.11	-0.094±0.60	0.69±0.24	1.45±0.41
48	0.07±0.10	0.20±0.50	0.96±0.63	1.39±0.90

Note: AHI means actual healthy index, PHI means predicted healthy index

ROS was employed as the response, which is the predictor in PLS model.

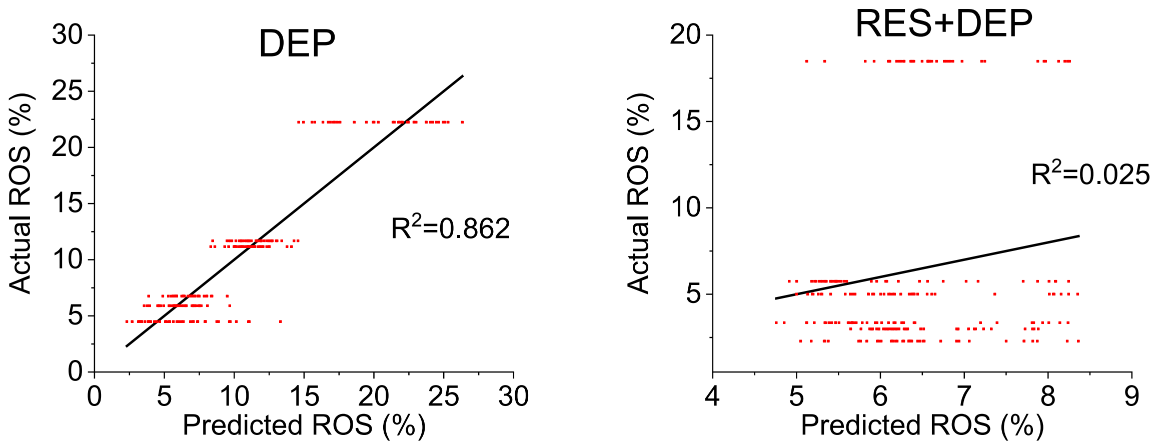

Figure S6: PLS model calculated from Raman data (240 spectra) of RES+DEP groups using ROS as the response.

Table S4: Validation of PLS model (ROS as response) for DEP and RES+DEP groups using a group of testing Raman data (8 spectra were randomly selected from each time point). Data are shown as mean ± SD.

Time (h)	DEP group		RES+DEP group	
	Actual ROS	Predicted ROS	Actual ROS	Predicted ROS
0	6.8±0.39	5.9±1.49	3.0±0.24	7.5±0.49
4	4.5±1.51	9.0±1.98	2.3±0.42	6.6±1.18
8	5.9±2.21	8.7±1.79	3.3±2.09	6.7±0.98
16	11.7±2.06	11.2±1.29	5.7±0.66	6.1±0.97
24	11.2±1.55	10.9±1.46	5.0±0.73	6.9±1.03
48	22.2±1.58	19.2±02.16	18.5±2.21	7.0±1.23

Fluorescence image

To detect potential alterations of cytoskeletal structures of SAEC treated with different DEP exposure times with or without RES, laser scanning confocal microscopy was applied to perform fluorescence and Differential Interference Contrast (DIC) imaging respectively, as shown in Figure S7. Confocal microscopy images show the fluorescence intensity decreases in a time-dependent manner for DEP group, suggesting cellular skeletons are disrupted by DEPs (Figure S7, rows 1 and 2). On the contrary, uniformly assembled cytoskeletal structures are clearly observed at all time points in the RES treatment group (Figure S7, row 4), indicating the presence of more cellular filamentous cytoskeleton and filopodia. These findings suggest that RES attenuated DEP-induced time-dependent down-regulation of cytoskeleton and cell architectures.

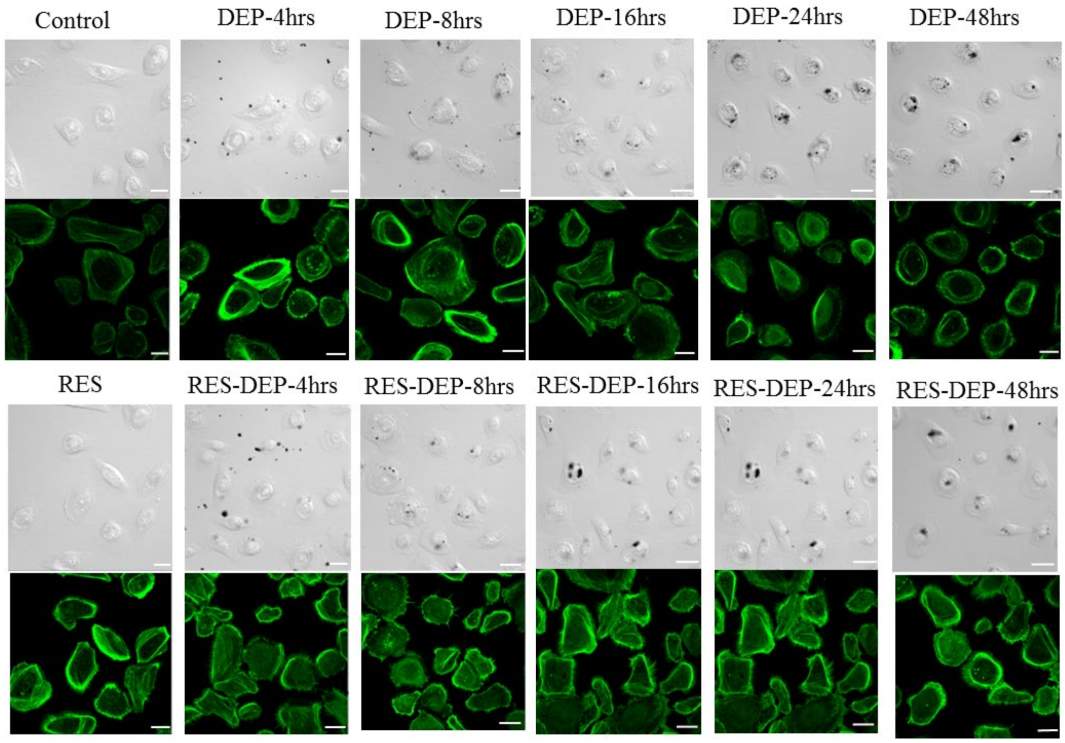

Figure S7: Differential interference contrast (DIC) images (row 1 and row 3) and corresponding confocal images (row 2 and row 4) of SAEC exposure to DEP with or without RES for different

time. DEPs (dark points in DIC images); cytoskeleton (fluorescein phalloidin, green in confocal images), scale bar=10 μm.

Cytokine and Chemokine analysis of RES treated DEPs-induced SAEC

Compared to the DEP group (Figure S8A), SAEC released five more varieties of cytokines and chemokines with RES treatment (Figure S8B). Upon RES treatment, SAEC released lower levels of GROα, IP-10, MCP-1, MCP-2 and RANTES. The other cytokines and chemokines (IL-1α, IL-6, IL-8, IL-10, IL-12p70, IL-13, IL-17, TNFα, Eotaxin, TARC and GM-CSF) levels were found to be close with and without RES treatment. Overall, it seems that cells resists to the abnormal external environment of RES and DEPs by expressing more varieties of mediators. And lower levels of pro-inflammatory responses (cytokines/chemokines) reveals that RES is protective against oxidative stress in a function of DEPs exposure time.

(A) DEP

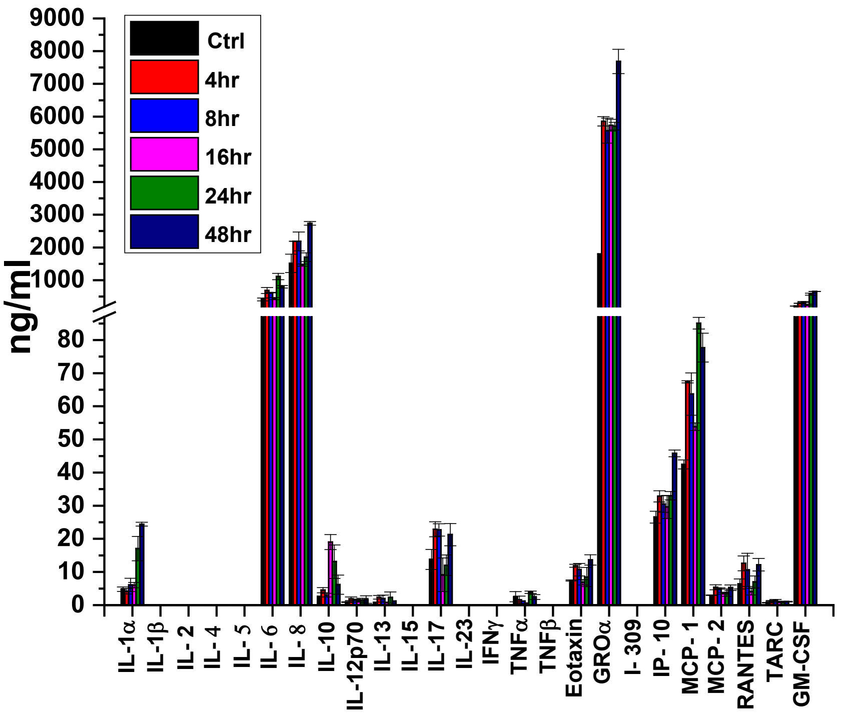

(B): RES+DEP

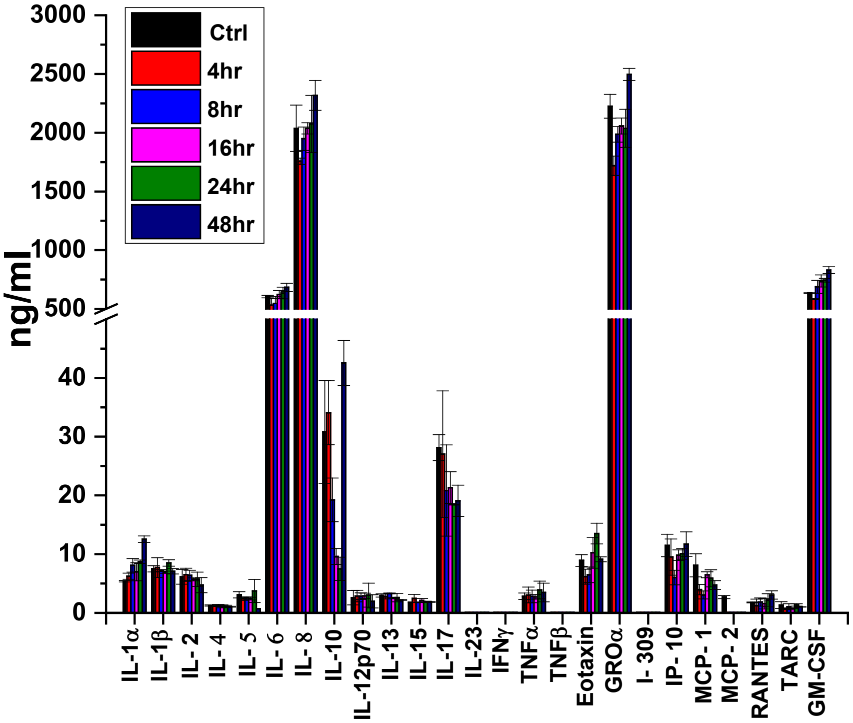

Figure S8. DEPs induced cytokines and chemokines release from SAEC without (A) and with (B) RES treatment. Barplots of IL-1α, IL-1β, IL-2, IL-4, IL-5, IL-6, IL-8, IL-10, IL-12p70, IL-13, IL-15, IL-17, IL-23, IFNγ, TNFα, TNFβ, Eotaxin, GROα, I-309, IP-10, MCP-1, MCP-2, RANTES, TARC and GM-CSF showed mean values of three independent experiments. Cells were exposed to 10μM RES and 10μg ml^{-1}DEP for 0h (without DEP treatment), 4h, 8h, 16h, 24h and 48h before measurement. Error bars are standard deviation of the mean.

Phenylalanine (1006 cm^{-1}) peak were employed as the healthy index, which is the predictor in PLS model. The training spectra were selected based on cell, which means randomly select three cells as the training group (each cell has 12 spectra) and the other one cell was used as testing.

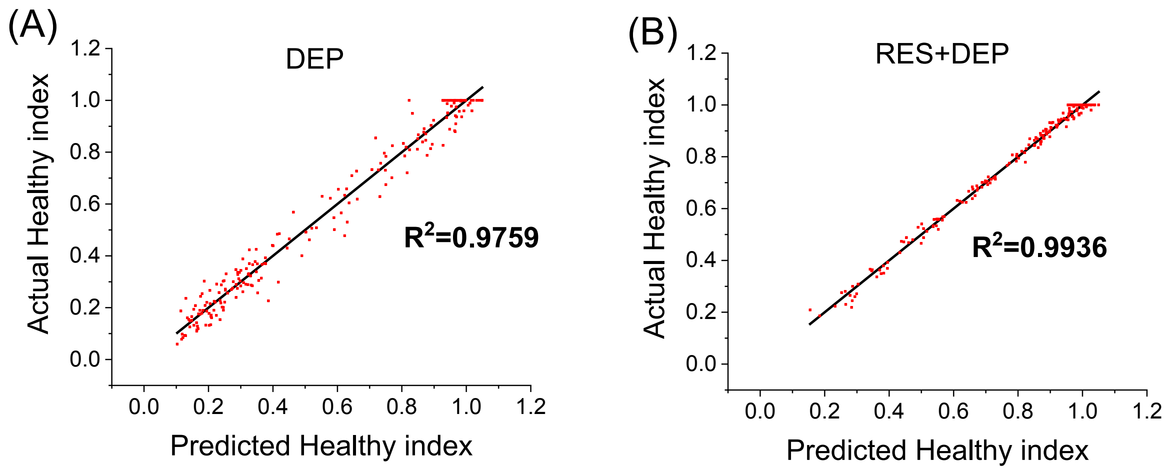

Figure S9: Partial Least square regression model of DEP (A) and RES+DEP (B) group, using phenylalanine (1006 cm^{-1}) intensity as healthy index (predictor) randomly selecting spectra on three cells in each time point (216 spectra).

Table S5: Validation of PLS model using Raman data on one cell (12 spectra of each time point)

	DEP group		RES+DEP group	
Time (h)	AHI	PHI	AHI	PHI
0	0.97±0.05	0.97±0.03	0.95±0.09	0.88±0.10
4	0.45±0.24	0.41±0.22	0.61±0.27	0.67±0.33
8	0.59±0.23	0.60±0.21	0.66±0.31	0.69±0.34
16	0.25±0.13	0.25±0.12	0.56±0.33	0.56±0.32
24	0.43±0.29	0.45±0.30	0.64±0.32	0.65±0.31
48	0.31±0.28	0.29±0.29	0.60±0.33	0.62±0.32

Note: AHI means actual healthy index, PHI means predicted healthy index

Morphological observation of SAEC and DEP under SEM

SEM images were taken with Hitachi S-4000. Figure S10 shows representative SEM images of control untreated SAEC and DEPs treated SAEC. DEPs can be observed on treated SAEC in SEM images (red arrow, Figure.S10D). The size of particles adherent to cell surface ranges from nanometer to micrometer.

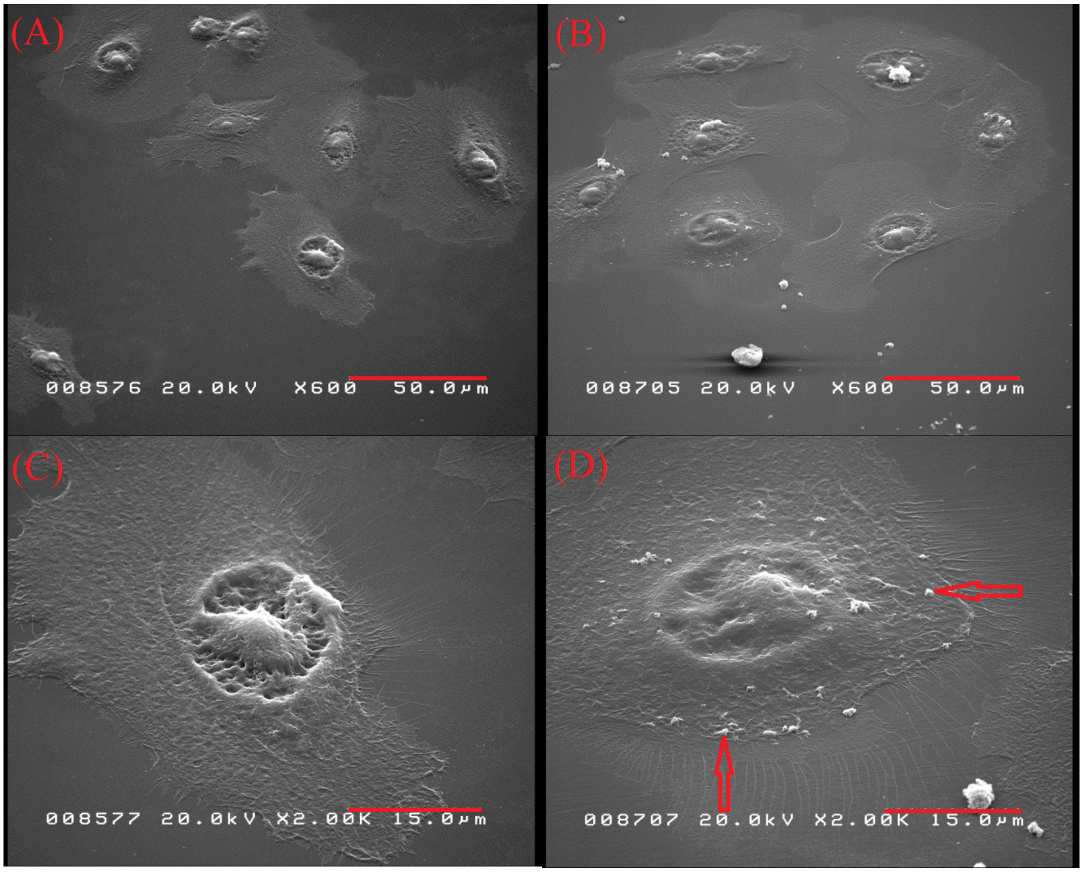

Figure S10. SEM images of control SAEC (a, c) and DEPs treated SAEC (b, d). Scale bar in A and B =50 μm, scale bar in C and D= 15 μm

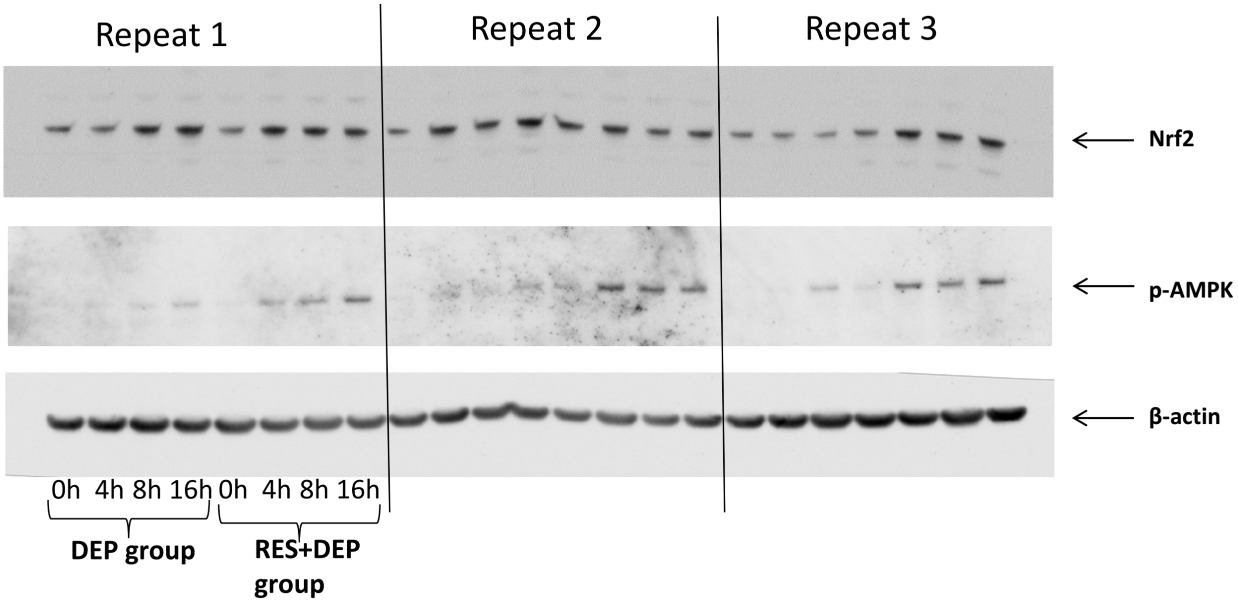

Figure S11: uncropped Western blot images of Nrf2, p-AMPK and β-actin.
